# Targeted metabolomics reveals the impact of glucose and pyruvate on energy metabolism and storage potential of stallion spermatozoa

**DOI:** 10.1007/s11306-025-02393-2

**Published:** 2026-03-28

**Authors:** Laura Becerro-Rey, Francisco Eduardo Martín-Cano, Antonio Silva-Rodríguez, Cristina Ortega-Ferrusola, Eva da Silva-Álvarez, Cruz Gil, Fernando J. Peña

**Affiliations:** 1 Laboratory of Equine Reproduction and Equine Spermatology, Veterinary Teaching Hospital, Universidad de Extremadura, Avenida de la Universidad s/n, 10003 Cáceres, Spain; 2 Facility of Innovation and Analysis in Animal Source Foodstuffs, Universidad de Extremadura, Cáceres, Spain

**Keywords:** Horse, Spermatozoa, Glucose, Pyruvate, Metabolism

## Abstract

**Introduction:**

Oxidative phosphorylation is the main source of ATP for the stallion spermatozoa. Consequently, metabolites that favor mitochondrial function are receiving increased interest. However, glycolysis itself may be the major source of pyruvate and acetyl-CoA.

**Objective:**

To determine the contribution of glycolysis to feed the tricarboxylic acid cycle to generate the reducing equivalents for the electron transport chain.

**Methods:**

We stored stallion spermatozoa in the presence of different concentrations of glucose and pyruvate (1mM glucose /1mM pyruvate, 1mM glucose /10 mM pyruvate, 40 mM glucose / 1 mM pyruvate, 40 mM glucose /10 mM pyruvate, 67 Mm glucose / 1 mM pyruvate and 67 mM glucose /10 mM pyruvate). We performed targeted metabolomics using UHPLC-MS/MS, as well as several flow cytometry and computer-assisted motility assays, to investigate sperm function during storage.

**Results:**

Pyruvate 10 mM improved the efficiency of glycolysis in the 40 mM glucose media. This improvement may be related to the action of lactate dehydrogenases as revealed by relative changes in lactate and pyruvate in this group. Interestingly, the TCA cycle is fed through glutamine and glutamate, and 10 mM pyruvate improves the efficiency of TCA in a 67 mM glucose extender. Lower methylglyoxal (*P* < 0.05) and higher levels of GSH (*P* < 0.01) were observed when the 1 mM glucose extender was supplemented with 10 mM pyruvate. The kinematic efficiency (*P* < 0.05) was higher in the low glucose media.

**Conclusion:**

Glucose probably contributes to stallion sperm metabolism feeding the TCA cycle, and aerobic glycolysis may play a major role in sperm functionality.

**Supplementary Information:**

The online version contains supplementary material available at 10.1007/s11306-025-02393-2.

## Introduction

The equine semen trade is essential for the industry, facilitating genetic progress by providing access to diverse stallions worldwide while enhancing biosecurity (Clulow & Gibb, 2022). It offers breeding flexibility and serves as the foundation for both in vivo and in vitro embryo production, which significantly supports the advancement of the industry and its economy (Clulow & Gibb, 2022). Recent developments in the study of stallion spermatozoa biology are altering various paradigms deeply embedded in clinical practice. The prevailing view regarding the role of oxidative phosphorylation as the primary source of ATP in stallion spermatozoa (Davila et al., 2016; Gibb et al., 2014) challenges the formulation of most current commercial extenders in use and the interpretation of reactive oxygen species. Furthermore, recent research highlights the toxic potential of supraphysiological concentrations of glucose in most commercial extenders (Martin-Cano et al., 2024b; Ortiz-Rodriguez et al., 2021). Equine scientists are re-evaluating the formulation of existing semen extenders (Hernandez-Aviles et al., 2020) and the roles of lactate and pyruvate as carbon sources (Darr et al., 2016; Ramirez-Agamez et al., 2024). Several studies, including proteomics, underscore the significance of sperm metabolism and the abundance of metabolic proteins in stallion spermatozoa (Gaitskell-Phillips et al., 2021, 2023; Griffin et al., 2019, 2022; Pena et al., 2024c). The study of sperm metabolism is gaining momentum from both a mechanistic and basic science perspective, as well as a means to enhance current reproductive biotechnologies (Albarracin et al., 2004; Jones, 1997; Ortiz-Rodriguez et al., 2024; Pena et al., 2022a, b; Sanchez-Rodriguez et al., 2022; Simonik et al., 2025; Van de Hoek et al., 2024).

Lactate and pyruvate emerged as key metabolites in the spermatozoa, and research on sperm metabolism is experiencing a renewed interest in spermatology (Darr et al., 2016; Hereng et al., 2011; Martin-Cano et al., 2024a; Paventi et al., 2015; Pena et al., 2024b; Reynolds et al., 2017; Schmidt et al., 2024). Although the beneficial effect of lactate and pyruvate has been largely attributed to their role as substrates for the TCA cycle, other mechanisms have been proposed. Recent evidence indicates that pyruvate’s beneficial effect primarily derives from its reduction to lactate (Hereng et al., 2011; Pena et al., 2025). The action of sperm lactate dehydrogenases (LDH) regenerates NAD^+^ by reducing pyruvate to lactate. The NAD^+^ is an essential cofactor necessary for efficient glycolysis and is also essential in many reactions in the TCA cycle (Becerro-Rey et al., 2024b; Martin-Cano et al., 2024a). This production of lactate under high oxygen concentration is termed aerobic glycolysis or Warburg effect, described initially in cancer cells (Warburg et al., 1927). Still, recent research from our laboratory revealed the important role of LDH for sperm function (Becerro-Rey et al., 2025; Pena et al., 2025), suggesting the presence of a similar mechanism in the spermatozoa and the importance of the regeneration of NAD^+^ through the isoform C of LDH (Becerro-Rey et al., 2025). Given this evidence, we aimed to establish how the sources of energy in the extender may influence the longevity of stallion spermatozoa during extended conservation at room temperature. We hypothesized that pyruvate may improve sperm conservation during storage thanks to the conversion to lactate and concomitant regeneration of NAD^+^, improving overall metabolic function. This study aimed to disclose metabolic changes induced by different concentrations of glucose and pyruvate in the extender; moreover, its relations with sperm survival and functionality were studied using flow cytometry and metabolomic analysis using UHPLC/MS/MS, aiming to expand our current knowledge on sperm aging during conservation and thus obtain clues to improve current conservation media.

## Material and methods

### Reagents and media

Monochlorobimane (MCB), and all other chemicals were purchased from Sigma Aldrich (Madrid, Spain). All other reagents for flow cytometry were purchased from Thermo Fisher (Carlsbad, Ca USA). ViaKrome 808 Fixable Viability Dye was purchased from Beckman Coulter (Indianapolis, IN USA). For mass spectrometry analysis, O-benzylhydroxylamine hydrochloride (O-BHA) and N‑(3‑dimethylaminopropyl)‑N′‑ethylcarbodiimide hydrochloride (EDC), and methylglyoxal were supplied by Sigma-Aldrich (St. Louis, MO, USA). LC-MS grade formic acid and acetonitrile were purchased from Thermo Fisher Scientific (Pittsburgh, PA, USA). Ultra-pure deionized water (> 18.2MΩ·cm) was produced from a Millipore Milli-Q Gradient system (Millipore, Bedford, MA, USA).

### Semen collection and processing

Semen was collected from four fertile stallions maintained according to institutional and European animal care regulations (Law 6/2913 June 11th and European Directive 2010/63/EU). Ejaculates were collected using a pre-warmed, lubricated Missouri model artificial vagina following standard veterinary practices. After collection, the ejaculate was immediately evaluated for sperm motility and concentration and processed in the adjacent laboratory. Colloidal centrifugation (Morrell et al., 2011) was performed to remove dead spermatozoa and potential contaminating cells from the ejaculate. The pellet was re-extended to a final concentration of 25 × 10^6^ spermatozoa/ml in variants of Tyrode´s media containing different concentrations of glucose and/or pyruvate: 1mM glucose /pyruvate 1mM (G1), 1 mM glucose / 10 mM pyruvate (G1-10P), 40 mM glucose / 1 mM pyruvate (G40), 40 mM glucose /10 mM pyruvate (G40-10P), 67 mM glucose / 1 mM pyruvate (G67), and 67mM glucose /10 mM pyruvate (G67-10P). The pH was adjusted to 7.4, and the osmolarity was adjusted to 310 mOsm/kg. The recipe for the different media is provided in supplementary Table 1.

### Experimental design

Individual ejaculates were split and extended to a concentration of 25 × 10^6^ spermatozoa per mL, in different media. Tyrode´s media, and the different variations above described, G1, G1-10P, G40, G40-10P, G67, G67-10P. All the aliquots were stored at 22 °C, up to 96 h. Two hours after extension (day 1), and after 48 (day 2) and 96 h (day 4) of storage aliquots were taken for sperm analysis. The parameters studied were the following: motility and kinematics (CASA), viability, acrosome integrity, apoptotic changes, mitochondrial activity, production of reactive oxygen species, and Ca^2+^ dynamics using multiparametric flow cytometry.

### Computer-assisted sperm analysis (CASA)

Sperm motility and kinematics were assessed using a Computer Assisted Sperm Analysis (CASA) system (ISAS Proiser, Valencia, Spain) according to standard protocols used at our center (Ortega-Ferrusola et al., 2009). Semen samples were loaded in a Leja^®^ chamber with a depth of 20 μm (Leja, Amsterdam, The Netherlands) and placed on a stage warmed to 37 °C. The analysis was based on an evaluation of 60 consecutive digitized images, with a frame rate of 60 Hz, obtained using a 10x negative phase-contrast objective (Olympus CX 41). At least 500 spermatozoa per sample were analyzed in random fields. Spermatozoa VAP > 35 μm/s were considered motile. Spermatozoa deviating < 45% from a straight line were classified as linearly motile. Other parameters studied included curvilinear velocity (VCL µm/s) defined as the time-averaged velocity of a sperm head along its actual trajectory, the straight-line velocity (VSL µm/s), the velocity calculated along a straight line between the first and last points of the path, and velocity along the average path (VAP µm/s) as the time-averaged velocity calculated along the average path. Additionally, kinematic efficiency (Curvilinear velocity/beat cross frequency) was calculated as described in (Schmidt et al., 2024) and can be interpreted as the average point-to-point distance along the sperm path in micrometers, traveled per cross of the sperm head across the path.

### Flow cytometry

Flow cytometry was conducted according to published guidelines (Cossarizza et al., 2021) adapted to spermatozoa. The analyses were conducted using a Cytoflex^®^ S flow cytometer (Beckman Coulter) equipped with violet, blue, yellow, and red lasers and a Cytoflex^®^ LX equipped with ultraviolet, violet, blue, yellow, red, and infrared lasers. The instruments were calibrated daily using specific calibration beads provided by the manufacturer. Files were exported as FCS files and compensated and analyzed in Cytobank© Software (Beckman Coulter, Brea, CA, USA). Unstained, single-stained, and Fluorescence Minus One (FMO) controls, were used to determine compensations and positive and negative events, as well as to set regions of interest as described in previous publications by our laboratory (Gallardo Bolanos et al., 2014; Martin Munoz et al., 2015) following the recommendations published as minimum information about a flow cytometry experiment standard (MyFlowCyt) (Lee et al., 2008).

#### Multiparametric flow cytometry panel assessing viability, mitochondrial reactive oxygen species, relative changes in intracellular Ca^2+^, and acrosomal integrity

This panel was run in the Cytoflex LX (https://www.beckmancoulter.com) flow cytometer. A five-color panel was created to simultaneously assess a wide range of sperm components and functions, including viability, acrosomal integrity, mitochondrial reactive oxygen species, thiols, and intracellular Ca^2+^. Stallion spermatozoa were stained with ViaKrome 808 (Beckman Coulter C36628 2µL/sample; Ex/Em(nm) 854/878) for viability analysis, while acrosome integrity was determined using PNA Ax647 conjugate (1µL of a 0.25 µg/ml solution). Reduced thiols were determined using monochlorobimane (MCB, 10µM) (Barhoumi et al., 1995), while mitochondrial superoxide production was assessed using Mitosox Red (0.5µM) and intracellular Ca^2+^ concentration using Fluo4 (100nM). Samples were incubated in the dark at 37 °C for 20 min, then washed and resuspended in PBS and run in the flow cytometer.

#### Four-color panel display (live-dead, caspase 3, mitochondrial membrane potential, and production of ROS)

This panel was run in the Cytoflex S flow cytometer. Aliquots of stallion spermatozoa containing 5 × 10^6^ spermatozoa in PBS were stained with CellEvent (2µM, Ex 502 nm, Em 530 nm) for detection of caspases 3 and 7, Tetramethylrhodamine (TMRM; 100nM, Ex 548 nm, Em 574 nm ) for determination of mitochondrial membrane potential, CellRox deep red (2 µM; Ex 638 nm, Em 665 nm) and 5µL of Live/Dead violet solution for viability analysis (Ex 405 nm, Em 450 nm) and incubated for 30 min in the dark at 37 °C. Cells were washed twice, resuspended in PBS, and run in the flow cytometer.

### Targeted metabolomics analysis

Metabolomic analysis was performed following published guidelines (Sumner et al., 2007). Semen samples were washed twice in ice-cold phosphate-buffered saline (PBS; 600 g for 10 min, fixed-angle rotor, deceleration set to 1) at 4 °C; the sperm pellet was flash-frozen in liquid nitrogen and stored at −80 °C until extraction. Pellets containing 1 × 10^8^ spermatozoa were resuspended in 300 µL of Milli-Q water/methanol (1:1, v/v), sonicated on ice for 3 s using a Branson Digital Sonifier (20% amplitude), then centrifuged (swing out centrifuge, 6452 g, 4 °C, 3 min, precooled rotor). The resulting supernatant was transferred to autosampler vials for UHPLC-MS/MS analysis.

Chromatography was performed on an Agilent 1290 Infinity II UHPLC system (Agilent Technologies, Santa Clara, CA, USA) equipped with an automated multisampler and high-speed binary pump, fitted with an Agilent HILIC-Z column (2.1 × 100 mm, 2.1 μm particle size; part no. 820192-912). The column oven was held at 35 °C and the flow rate set to 0.4 mL/min; injection volume was 7 µL. Solvent A consisted of 10 mM ammonium acetate (pH 9.0) in Milli-Q water; solvent B was 10 mM ammonium acetate (pH 9.0) in Milli-Q water: acetonitrile (10:90, v/v). The gradient was programmed as follows: 0–5 mi, 98% B; 5–10 min, linear decrease to 60% B; 10–12 min, hold at 60% B; 12–15 min, return to 98% B and re-equilibration.

Mass spectrometric detection was carried out on an Agilent 6470 QqQ mass spectrometer with AJS-Dual ESI interface, operated in negative-ion MRM (multiple reaction monitoring) mode. Nitrogen served as a nebulizer, drying, sheath, and collision gas. Nebulizer pressure was 35 psi; drying gas flow was 12 L/min at 250 °C; sheath gas flow was 15 L/min at 350 °C. Capillary spray voltage was 3,500 V and fragmentor voltage 100 V. Multiple reaction monitoring (MRM) transitions (precursor→product m/z) and collision energies were optimized for each metabolite via direct infusion of standards; transitions are listed in Supplementary Table S2. A rigorous quality control protocol was applied throughout the analytical sequence. Initially, a blank of the solvent extract was injected to establish baseline conditions. This was followed by multiple blank injections to condition and stabilize the analytical system. To monitor potential carryover effects, blank samples were injected after every 20 sample injections. Additionally, a metabolite mix—comprising representative metabolites of interest—was injected at regular intervals (every 60 samples) to verify the stability and reliability of analyte detection across the entire batch.

Data were acquired with MassHunter Workstation Data Acquisition software (Rev. B.08.00) and processed using MassHunter Quantitative Analysis (Rev. B.07.00.201). Peak areas were normalized to total ion current (TIC) per run and, where applicable, to internal standards; normalized data were log₂-transformed prior to downstream statistical analysis (Deininger et al., 2011).

### UHPLC/MS measurement of methylglyoxal

The sperm pellet containing 100 × 10^6^ spermatozoa was resuspended in 300 µl of Milli-Q water and sonicated for 5 s. Immediately afterwards, it was centrifuged (swing-out centrifuge) at 6272 x g at 4 °C for 3 min, and the supernatant was subjected to a derivatization procedure. The derivatization reagent was O-BHA coupled to EDC, which catalyzed the derivatization reaction. This reagent is suitable for derivatizing acids, ketones, and aldehyde groups. The derivatization was carried out following the procedure for the analysis of short-chain fatty acids (Zeng & Cao, 2018) with modifications. Specifically, 100 µl of supernatant was incubated with 20 µl of 0.1 M BHA in MeOH and 20 µl of 0.25 M EDC in MeOH at 35 °C for 1 h. After incubation, 50 µl was diluted 20-fold in 50% methanol, and 500 µl of dichloromethane was added to liquid-liquid extraction. Finally, a volume of dichloromethane (containing the derivatized analytes) was evaporated until dry. The residue was reconstituted in 100 µl of 50% aqueous MeOH and vortexed briefly, and 5 µl was injected on UHPLC-MS/MS. The effectiveness of the process was evaluated. Calibration was carried out by a standard addition technique. Therefore, the standard for methylglyoxal was prepared in supernatant samples spiked with increasing amounts, following the same derivatization procedure. The derivatized methylglyoxal (BHA-MGly) was analyzed by UHPLC-MS/MS. An Agilent 1290 Infinity II UHPLC coupled with 6470 triple quadrupole (QqQ) (Agilent Technologies, Waldbronn, Germany) was used. The UHPLC was equipped with a built-in autodegasser, binary pump and column thermostat. A Zorbax C18 column, 100 mm x 2.1 mm, 1.8 μm (Agilent, CA) was used for the separation step at 25 °C. The LC-MS interface was ESI with a jet stream. Nitrogen was used as the nebulizing gas, drying gas, sheath gas, and collision gas. The mobile phase was composed of two solutions: A, aqueous with 0.5% formic acid, and B, acetonitrile with 0.5% formic acid. A binary gradient was applied with a flow rate of 0.4 ml/min: 0–5 min 20% B, 5–10 min linear increase from 20 to 100% B, and maintained until minute 12, followed by re-equilibration of the column up to minute 15. BHA-MGly was eluted at 6.8 min. The ionization source parameters, operating in positive polarity, were optimized by injecting 3 mg/l BHA-MGly. The highest sensitivity was obtained with the following ionization source parameters: drying gas temperature at 200 °C, nebulizer at 25 psi, drying gas flow at 12 L/min, sheath gas temperature and flow rate at 350 °C and 10 L/min, respectively, capillary voltage at 3500 V, and fragmentor at 110 V. The MRM conditions were optimized by injecting the same solution at different collision energies (CE). The transitions were from 283 to 91 (at 25 eV CE) and to 181.1 (at 10 eV CE) for the selected MRMs to determine sensitivity and selectivity. The quantification transitions were 283 to 91 for BHA-MGly.

### Statistical analysis

All experiments were repeated at least three times with independent biological replicates. The normality of the data was assessed using the Kolmogorov-Smirnov test. One-way ANOVA followed by Tukey multiple comparisons test as post hoc analysis was performed using GraphPad Prism version 10.00 for Mac, La Jolla California USA, www.graphpad.com. Differences were considered significant when, after correction for multiple comparisons, *P* < 0.05. Results are displayed as means ± SEM.

## Results

### The composition of the media impacts motility

**Fig. 1:**
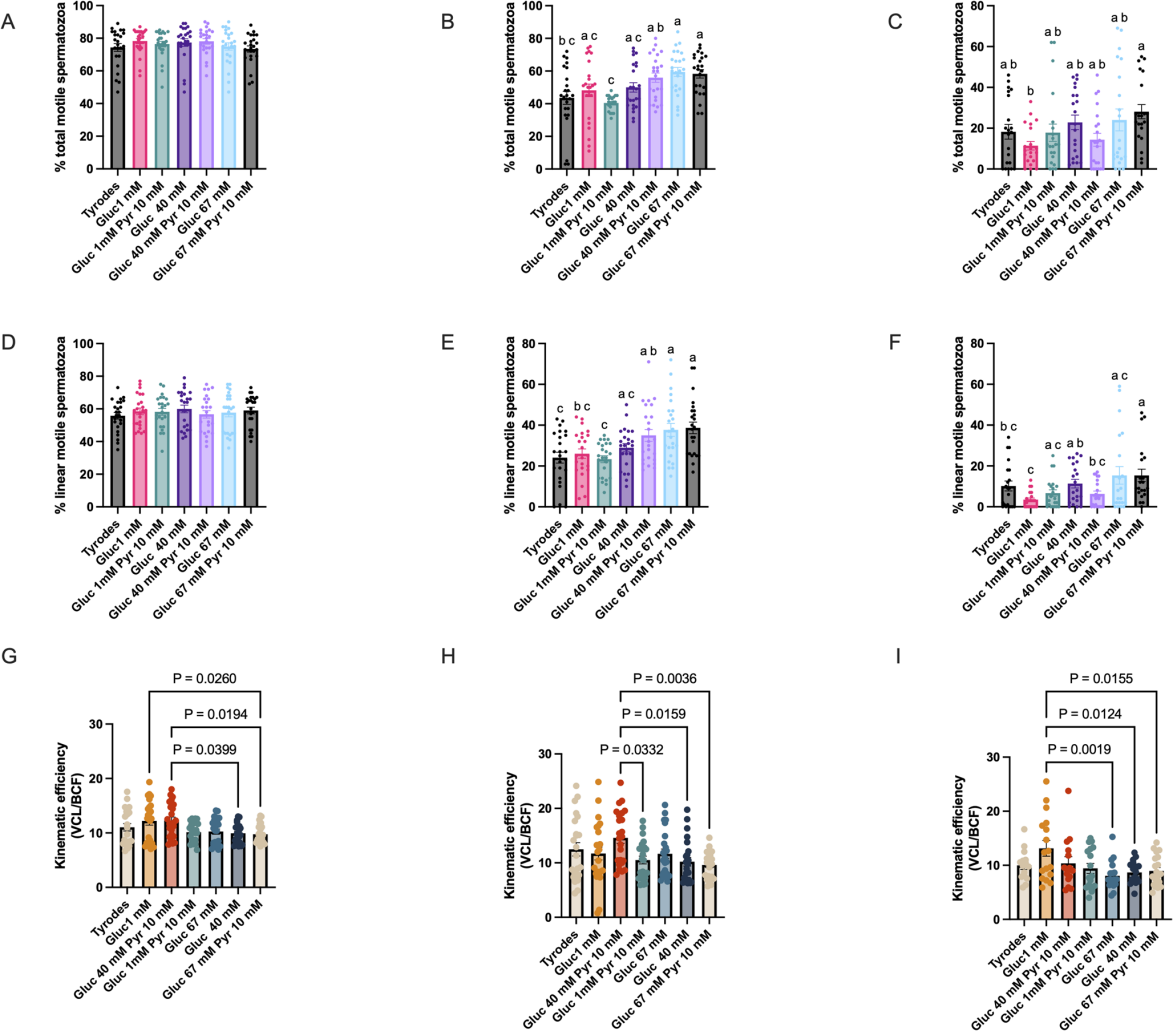
Effect of the concentrations of glucose (1, 40, and 67 mM) and pyruvate (1 and 10 mM) in the media on stallion sperm motility (total and linear) stored up to 96 h at room temperature. Stallion ejaculates were processed as described in material in methods and stored up to 96 h at r.t. in media with varying concentrations of glucose and pyruvate. Motility was determined using computer-assisted sperm analysis (CASA) shortly after extension and after 48 and 96 h of storage. The percentage of total motility is given in **A** (after extension) **B** (after 48 h of storage at r.t.) **C** (after 96 h of storage at r.t). The percentage of linear motile spermatozoa is given in **D** (after extension) **E** (after 48 h of storage at r.t.) **F** (after 96 h of storage at r.t). Results are given as means ± SE. a-c *P* < 0.01, a-b *P* < 0.05. Results are derived from 12 replicates, 4 different stallions, 3 replicates each. **G**-**I**: Effect of the concentrations of glucose (1, 40, and 67 mM) and pyruvate (1 and 10 mM) in the kinematic efficiency defined as the average point-to-point distance along the sperm path in micrometers traveled per cross of the sperm head across the path (measures the strength of every single flagellar stroke). Stallion spermatozoa were stored for up to 96 h at room temperature. Stallion ejaculates were processed as described in Materials and Methods and stored up to 96 h at r.t. in media with varying concentrations of glucose and pyruvate. Kinematic efficiency was determined using computer-assisted sperm analysis (CASA), applying the following formula: VCL/BCF shortly after extension (**G**) and after 48 (**H**) and 96 h of storage (**I**). Results are derived from 12 replicates, 4 different stallions, 3 replicates each

Although motility did not change concerning the media at the beginning of the incubation period, changes were evident after 48 and 96 h of storage (Fig. 1). After 48 h of incubation, a higher percentage of total and linear motility was observed in aliquots extended in the 40 G 10 P, G67, and G67-P10 (56.0 ± 2.8, 59.5 ± 2.7, and 58.3 ± 2.5, respectively for the percentages of total motility). After 96 h of storage at 22 °C, the highest percentage of total motile spermatozoa was observed in aliquots stored in the G67-10P medium (24.6 ± 3.6%) and the worst in the G1(10.0 ± 1.9%) (Fig. 1C; *P* = 0.003). The percentages of linear motile spermatozoa followed a similar trend, with the highest results after 48 h of storage observed in the G40, G40-P10, G67 and G67G-P10 (Fig. 1 E).

### The media’s composition impacts stallion spermatozoa’s kinematic efficiency

The media composition significantly impacted kinematic efficiency (KE) at every time considered. This parameter (Schmidt et al., 2024) is an estimate of the strength or energy available for the spermatozoa, combining in one, different aspects of sperm motility and kinematics. At the beginning of the storage period, higher kinematic efficiency was observed in low glucose media G1 and G1-10P, with KEs of 11.0 ± 0.6 μm and 12.2 ± 0.7 μm, which were superior to the KEs observed in the high glucose media G67 and G67-P10 9.9± 0.3 μm and 9.7 ± 0.3 μm respectively (*P* = 0.026; *P* = 0.019; *P* = 0.039; Fig. 1G). After 48 h of storage, higher KE was observed in the G1-10P media (14.6 ± 1.0), which was superior to the G40 (10.5 ± 0.7; *P* = 0.03), 67G (10.2 ± 0.8 *P* = 0.01) and G67-10P mM (9.6 ± 0.5; *P* = 0.03; Fig. 1H). The same tendency was observed after 96 h of storage (Fig. 1G), with the highest KE observed in the G1 group, which was superior to the G40-10P, (*P* = 0.002), the G67 mM glucose (*P* = 0.01) and G67-10P (*P* = 0.01).

### The composition of the media influenced Ca^2+^ concentration in the population of live spermatozoa

**Fig. 2:**
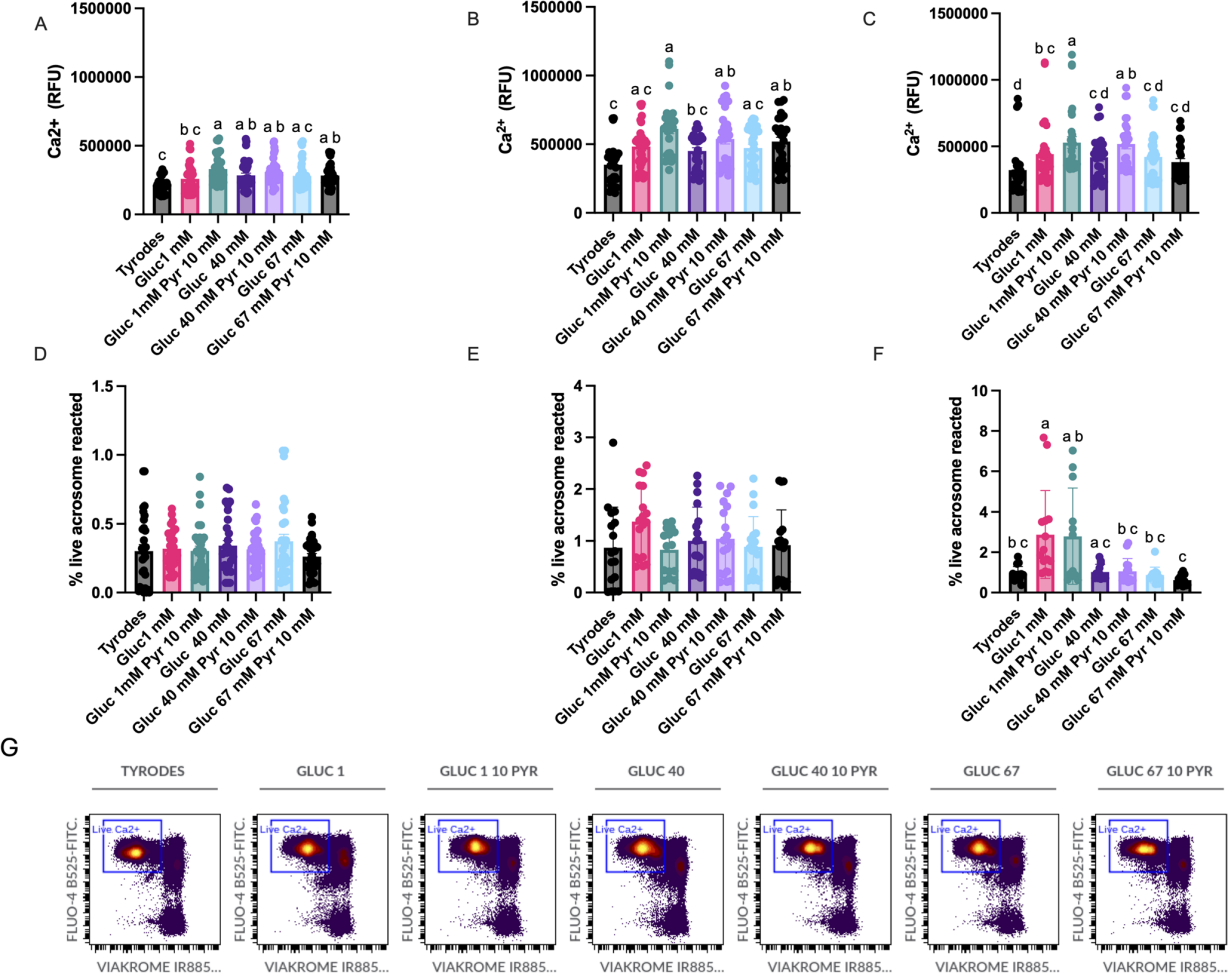
Effect of the concentrations of glucose (1, 40, and 67 mM) and pyruvate (1 and 10 mM) in the media on Ca^2+^ content in viable spermatozoa and capacitation (determined by the presence of the spontaneous acrosome reaction in viable spermatozoa) stored up to 96 h at room temperature. Stallion ejaculates were processed as described in material in methods and stored up to 96 h at r.t. in media with varying concentrations of glucose and pyruvate. Ca^2+^ content in viable spermatozoa is given in **A** (after extension) **B** (after 48 h of storage at r.t.) **C** (after 96 h of storage at r.t). The percentage capacitated (acrosome reacted) is given in **D** (after extension) **E** (after 48 h of storage at r.t.) **F** (after 96 h of storage at r.t). Results are given as means ± SE. a-c *P* < 0.01, a-b *P* < 0.05. Results are derived from 12 replicates, 4 different stallions, 3 replicates each. Data with different superscripts differ statistically a-b *P* < 0.05, a-c *P* < 0.01 **G** representative cytograms are given

Media composition had a major effect on the relative intracellular Ca^2+^ concentration (Fig. 2). From the beginning of the storage period, Ca^2+^ content in the population of live spermatozoa, was lower in the spermatozoa extended in the Tyrode’s basal media (Fig. 2). Particularly, the intracellular Ca^2+^ concentration was higher in the G1-P10 and G40 and G40-P10 respect Tyrode’s (Fig. 2A; *P* < 0.0001). This tendency was maintained after 48 h, with once again the intracellular Ca^2+^ concentration higher in the G1-10P (Fig. 2B; *P* < 0.0001). After 96 h of storage, Ca^2+^ concentrations were higher in all the media, except for the Tyrode’s basal media (Fig. 2C; *P* < 0.01).

### The composition of the media did not affect sperm viability, but low concentrations of glucose favored capacitation after 96 h of storage

Although a decrease in sperm viability (live-acrosome reacted spermatozoa) was observed after 96 h of storage, no differences were observed concerning the media in which the spermatozoa were extended (Fig. 2D-E). However, after 96 h of storage, more live, acrosome-reacted spermatozoa were observed in media with low glucose concentration, G1 and G1-10P (Fig. 2F, *P* < 0.01).

### Effect of media composition on mitochondrial membrane potential (ΔΨm), and production of reactive oxygen species in the population of viable spermatozoa during storage

**Fig. 3:**
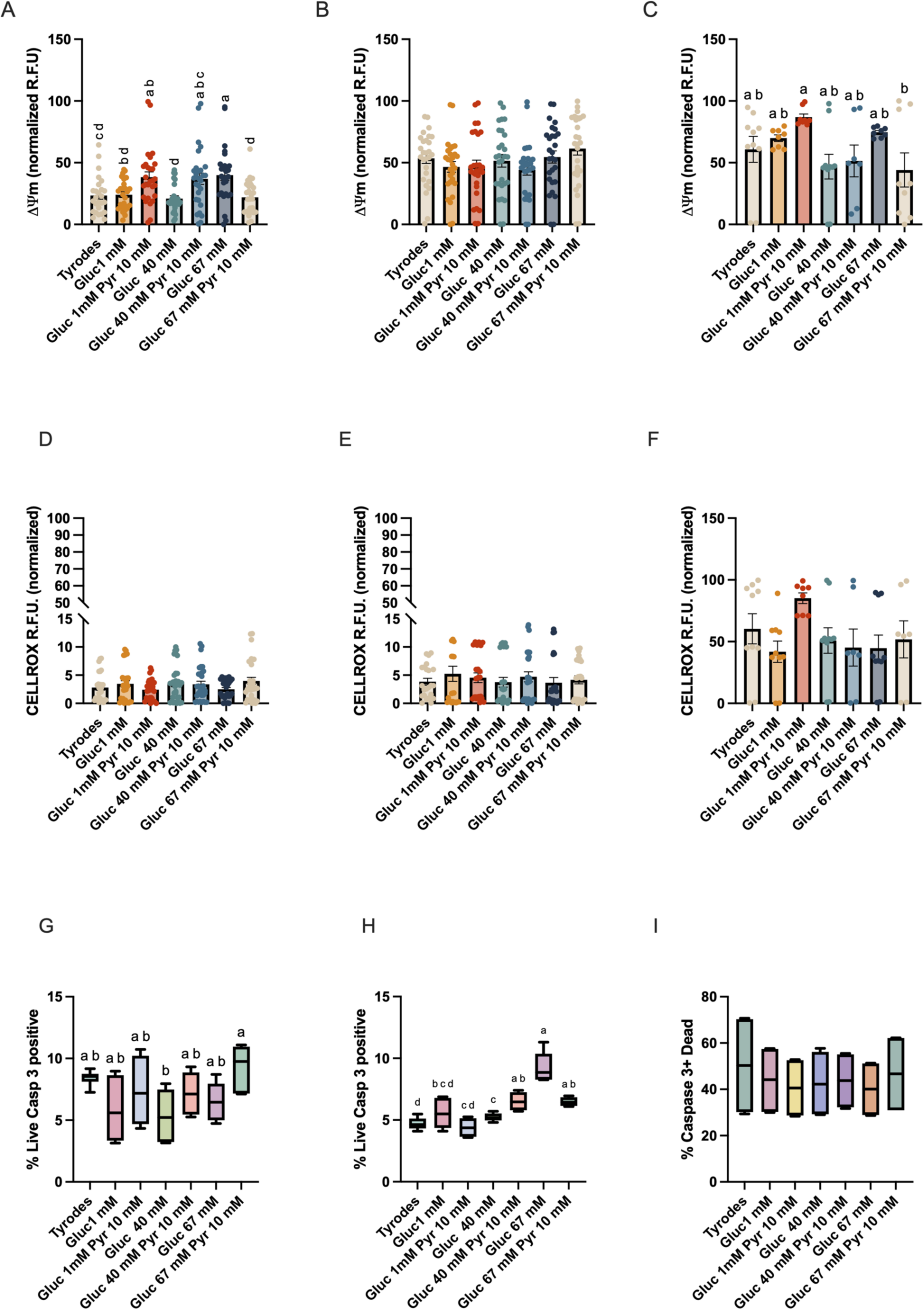
Effect of the concentrations of glucose (1, 40, and 67 mM), and pyruvate (1 and 10 mM) in the media on mitochondrial membrane potential (ΔΨm), production of reactive oxygen species ROS, and percentage of live spermatozoa showing caspase 3 activity in viable spermatozoa stored up to 96 h at room temperature. Stallion ejaculates were processed as described in material in methods and stored up to 96 h at r.t. in media with varying concentrations of glucose and pyruvate. The mitochodrial activity in viable spermatozoa is given in **A** (after extension) **B** (after 48 h of storage at r.t.) **C** (after 96 h of storage at r.t). The production of superoxide anion is given in **D** (after extension) **E** (after 48 h of storage at r.t.) **F** (after 96 h of storage at r.t). The percentage of viable spermatozoa showing active caspases 3 and 7 is given in **G** (after extension), **H** (after storage for 48 h at r.t) and **I** (after storage for 96 h at r.t). Results are given as means ± SE. a-d *P* < 0.01, a-b *P* < 0.05. Results are derived from 12 replicates, 4 different stallions, 3 replicates each

**Fig. 4:**
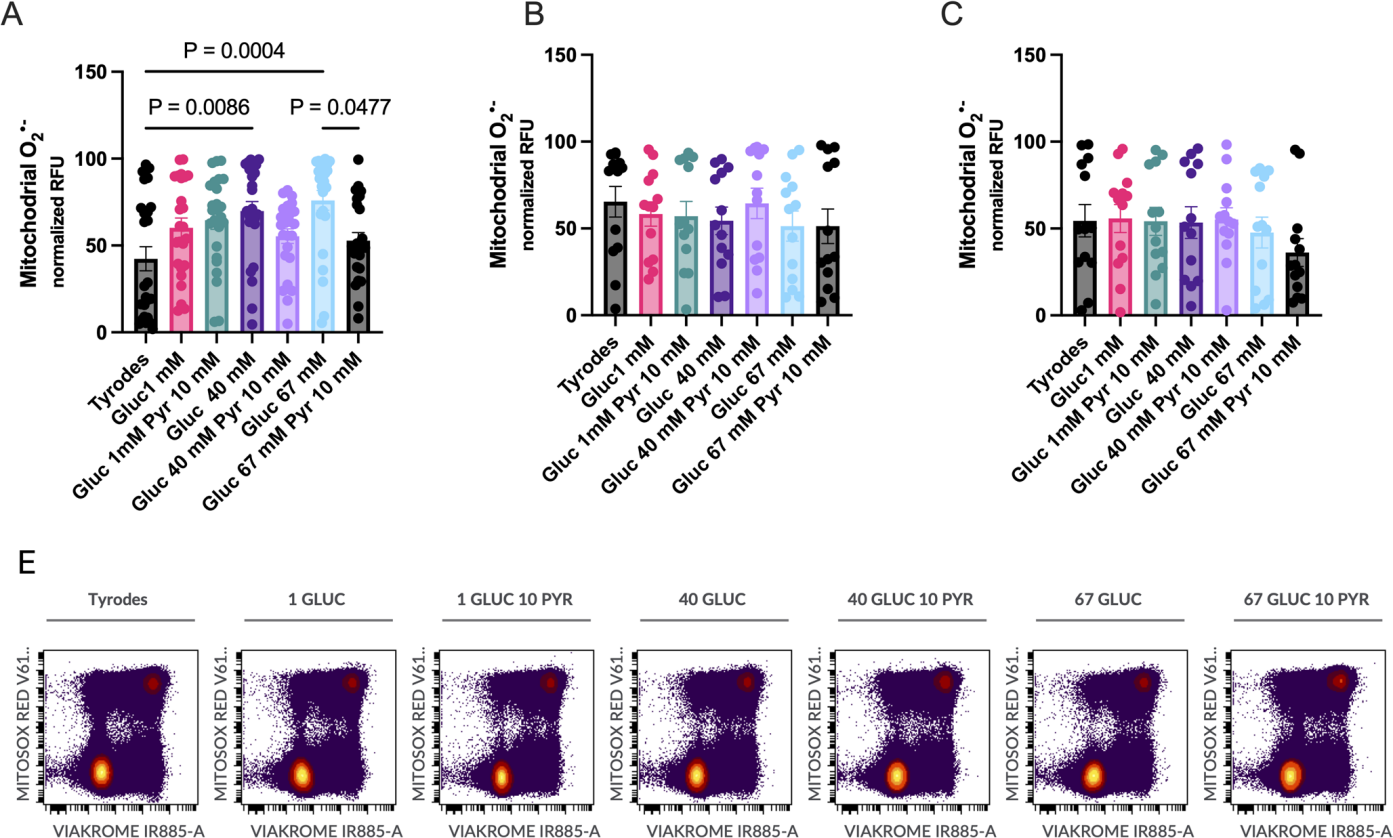
Effect of the concentrations of glucose (1, 40, and 67 mM), and pyruvate (1 and 10 mM) in the media on mitochondrial production of superoxide (O_2_^•−^), using flow cytometry in viable spermatozoa stored up to 96 h at room temperature. Stallion ejaculates were processed as described in material in methods and stored up to 96 h at r.t. in media with varying concentrations of glucose and pyruvate. The production of O_2_^•−^ was determined using flow cytometry shortly after extension and after 48 and 96 h of storage. The mitochondrial production of superoxide is given in **A** (after extension) **B** (after 48 h of storage at r.t.) **C** (after 96 h of storage at r.t). Results are given as means ± SE. Results are derived from 12 replicates, 4 different stallions, 3 replicates each. In **E** representative cytograms are given

At the beginning of the storage period, media composition impacted mitochondrial activity (Fig. 3A). Spermatozoa stored in the G40-10P, G1-10P and G67 displayed more intense mitochondrial activity, while the lowest was shown in spermatozoa stored in the G40 (Fig. 3A; *P* < 0.01). After 48 h of storage, there were no differences in mitochondrial activity between media, but after 96 h of storage at room temperature, differences were again evident. After 96 h of incubation, the more intense mitochondrial activity was observed in the spermatozoa stored in the G1-10P, while the poorest was in spermatozoa stored in the G67-10P media (Fig. 4C; *P* < 0.05). Concerning the production of reactive oxygen species, there were no effects of the media. However, the production of reactive oxygen species increased independently of the media after 96 h of storage (Fig. 3D- F).

### Effect of media composition on the activation of caspase 3 in the population of live spermatozoa

Media composition impacted the percentages of live spermatozoa showing caspase 3 activity after 48 h of incubation (Fig. 3H). At the beginning of the incubation period, the percentage of live spermatozoa with active caspases was higher in the 67G-10P group in comparison with the G40 group (Fig. 3G; *P* < 0.05). After 48 h of storage, the percentage of spermatozoa showing active caspases was higher in the G67 group (Fig. 3H; *P* < 0.01).

### Effect of media composition in the production of mitochondrial superoxide anion (O_2_^•−^) in the viable population of spermatozoa

At the beginning of the storage period, differences in the production of mitochondrial O_2_^•−^ were observed, but disappeared after 48 h of storage. The production of O_2_^•−^ was higher in viable spermatozoa stored in the G40 and G67 (Fig. 4; *P* < 0.01) than those stored in Tyrode’s basal media. Interestingly, the addition of pyruvate 10 mM to the glucose 67 mM media reduced the production of mitochondrial O_2_^•−^ (Fig. 4A; *P* < 0.05).

## Energetic metabolism

Changes were investigated throughout the storage period in different media, and the TCA Cycle, Warburg effect, and glycolysis were investigated. Methylglyoxal production was also monitored using UHPLC/MS/MS, while changes in reduced glutathione were monitored using flow cytometry.

### Glycolysis

Glycolysis was monitored based on the phosphorylation of glucose, production of fructose 1,6-bisphosphate, and phosphoenolpyruvate. Glucose and pyruvate concentration and time impacted all these metabolites, suggesting modifications in glycolysis (Supplementary Fig. 1). In media with G1, and media with G1-10P, the phosphorylation of glucose remained stable. On the extender containing G40, the phosphorylation of this hexose increased gradually over time, reaching a peak after 96 h of storage (*P* < 0.05), while in G40-10P it decreased at 96 h of storage. In the media containing G67-10P, glucose phosphorylation remained constant, independent of the storage period.

The more evident changes in the glycolysis intermediate fructose 1, 6, biphosphate occurred in spermatozoa stored in the G1 extender, with an increase after 96 h of storage (*P* < 0.01). The relative content of phosphoenolpyruvate in the spermatozoa was lower in all media containing 10 mM pyruvate.

### Pentose phosphate pathway (PPP)

Two intermediates of the PPP were studied, ribulose 5-phosphate and 6-phosphogluconate (Supplementary Fig. 2). The main effect was observed in both of the G1 and G1-10P media, in which the presence of 10 mM pyruvate caused a significant reduction in comparison with the G1 (*P* < 0.01).

### Warburg effect or aerobic glycolysis

**Fig. 5:**
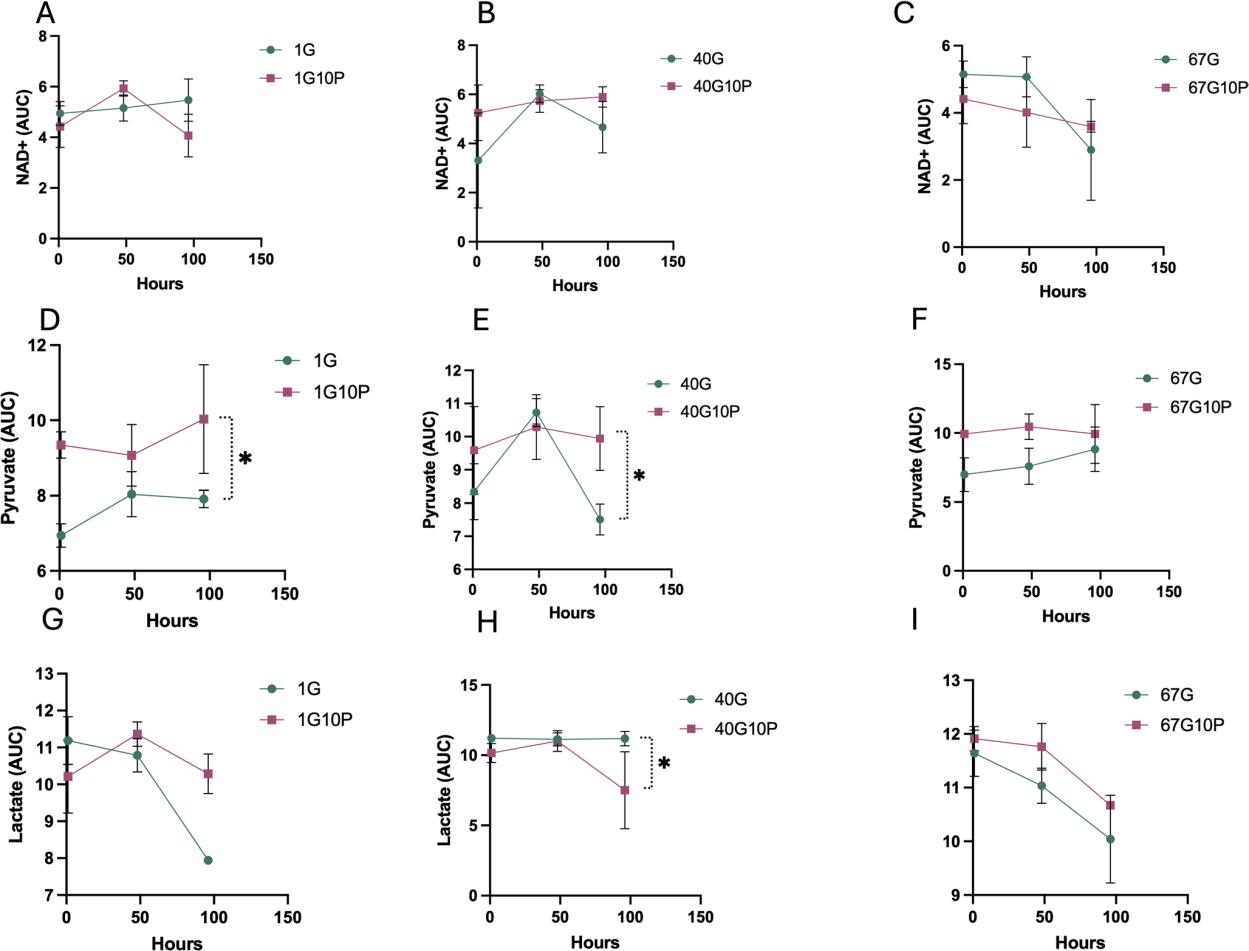
Changes in pyruvate, lactate and NAD^+^, stallion ejaculates were processed as described in material and methods and stored up to 96 h at r.t. in media with different concentrations of glucose and pyruvate: 1mM glucose 1 mM pyruvate (1G), 1mM glucose 10 mM pyruvate (1G 10P), 40 mM glucose 1 mM pyruvate (40G), 40 mM glucose 10 mM pyruvate (40G10P), 67mM glucose 1 mM pyruvate (67G) and 67 mM glucose and 10 mM pyruvate (67G10P). Data represent log transformed area under the curve data derived from UHPLC-MS QqQ and are given as means ± SE. Results are derived from 12 replicates, 4 different stallions, 3 replicates each. * *P* <0.05

The composition of the extender influenced the relative amounts of NAD^+^, lactate, and pyruvate. In the extender containing G40-10P NAD^+^ remained stable along the storage period; in this extender, intracellular pyruvate was higher (Fig. 5E; *P* < 0.05), while intracellular lactate decreased (Fig. 5H; *P* < 0.05) after 96 h of storage.

### Krebs cycle

**Fig. 6:**
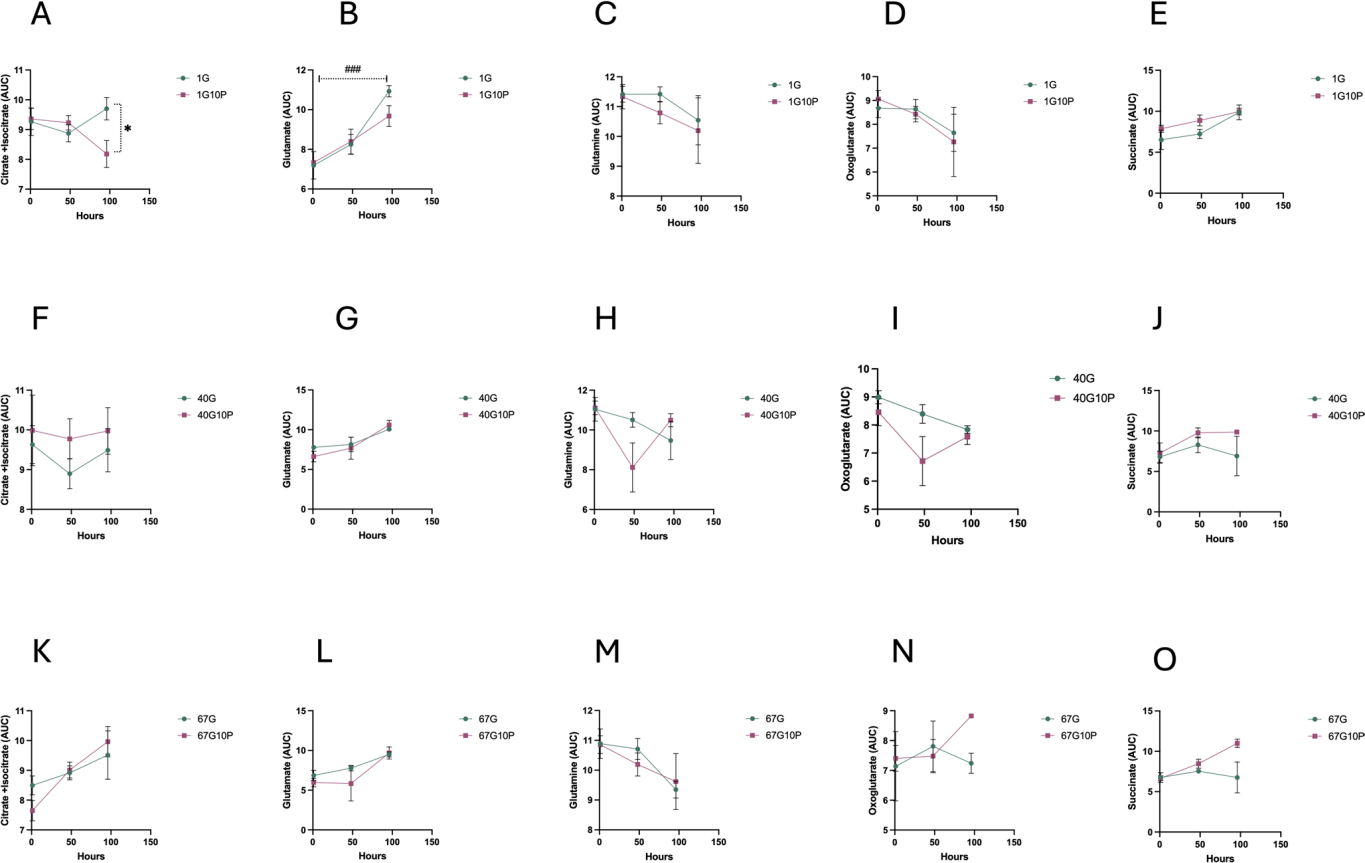
Tricarboxylic acid cycle (TCA) metabolomics stallion ejaculates were processed as described in material and methods and stored up to 96 h at r.t. in media with different concentrations of glucose and pyruvate: 1mM glucose 1 mM pyruvate (1G), 1mM glucose 10 mM pyruvate (1G 10P), 40 mM glucose 1 mM pyruvate (40G), 40 mM glucose 10 mM pyruvate (40G10P), 67mM glucose 1 mM pyruvate (67G) and 67 mM glucose and 10 mM pyruvate (67G10P). Data represent log transformed area under the curve data derived from UHPLC-MS QqQ and are given as means ± SE. Results are derived from 12 replicates, 4 different stallions, 3 replicates each. * *P* < 0.05, *P* < 0.05, ### *P* < 0.001

Different metabolites of the TCA cycle were monitored, including citrate, isocitrate, glutamine, glutamate, oxoglutarate, and succinate. Relative concentrations of citrate and isocitrate were reduced in the G1-10P extender in comparison with the G1 (Fig. 6A; *P* < 0.05). Glutamate increased over time in both media containing glucose 1mM (Fig. 6B; *P* 0,001). Citrate, isocitrate, and succinate tended to be higher in the G40-10P while oxoglutarate was reduced (Fig. 6 F-P).

### Production of methylglyoxal and concentrations of GSH

Changes in the relative levels and GSH content were monitored. Overall levels of methylglyoxal remained lower in media supplemented with pyruvate 10 mM, being significantly lower in the G1-10P media (*P* < 0.05; Supplementary Fig. 3). Interestingly, in this group, GSH concentration was also higher (*P* < 0.01).

## Discussion

In the present study, we investigated the effect of glucose concentration in the presence of basal concentrations of pyruvate (1 mM) or higher concentrations (10 mM) on sperm survival and functionality during storage at room temperature. Additionally, we monitored changes in the intracellular metabolome under these storage conditions. In the first hours of storage, no significant effects were observed in motility and membrane integrity. However, changes in intracellular Ca^2+^, mitochondrial membrane potential, kinematic efficiency, mitochondrial production of O_2_^•−,^ and percentage of live spermatozoa showing active caspase 3, were observed in the first hours of storage.

These findings suggest that changes induced by the metabolic substrates present in the media occur rapidly. After 48 h of storage at r.t. (21 °C), changes in most of the sperm parameters studied became evident. Motility was higher in media containing high glucose concentrations (67 mM and 40 mM). However, statistically significant differences were only observed between the G1-10P and the G67 and G67-10P. After 96 h of storage, statistical differences were only significant between the G1 and G67-10P extender. We included the parameter of Kinematic efficiency. This parameter has been recently introduced in the study of mouse sperm metabolism; it is believed to be a trustworthy indicator of overall sperm functionality and metabolic efficiency (Schmidt et al., 2024). This parameter revealed that metabolic efficiency may be superior in spermatozoa extended in low glucose extenders; this finding is in line with recent reports of our laboratory (Ortiz-Rodriguez et al., 2021), indicating higher functionality in sperm stored in low glucose media, related to improved metabolism and reduced production of toxic oxoaldehydes.

A clear effect was observed in the percentage of live acrosome-reacted spermatozoa in the low 1 mM glucose group, while no changes were seen in the percentages of intact membranes. A significant increase in the percentage of live caspase 3-positive spermatozoa was observed in spermatozoa extended in the G67 media. In this study, we conducted a detailed metabolic analysis of spermatozoa stored in different media. The efficiency of glycolysis improved, as indicated by the reduction in the relative amounts of phosphoenolpyruvate in the G1-10P extender at the start of the incubation period and after 48 h of storage; this corresponds to the improved KE seen during this period. Additionally, the relative concentrations of pyruvate and lactate were higher in the 1G-10P group. This may reflect different situations: the reduction of pyruvate to lactate with increased NAD^+^, favoring glycolysis through the action of isoforms A and C of LDH, the conversion of pyruvate to lactate through isoform B, and the increased activity of pyruvate dehydrogenase (PDH), converting pyruvate to Acetyl-CoA and feeding the TCA cycle. The presence of these three isoforms in stallion spermatozoa has been recently described in our laboratory (Becerro-Rey et al., 2025). The production of the toxic oxoaldehyde methylglyoxal was reduced in the presence of 10 mM pyruvate in the low glucose media, and levels of reduced glutathione were higher, suggesting that pyruvate may enhance redox regulation, probably balancing the NADH/NAD^+^ redox pair. This may indicate that the positive effect of pyruvate relies on its role in supporting effective glycolysis, favoring NAD^+^ availability (NAD^+^ remained stable in the G40-10P during the storage period) and not necessarily fueling OXPHOS, which may be primarily fueled through endogenous substrates, the production of which is favored in the presence of exogenous pyruvate (Balbach et al., 2020; Becerro-Rey et al., 2025). Interestingly, this metabolic plasticity is likely acquired after ejaculation (Balbach et al., 2023). The findings observed in the G40-10P further support this hypothesis; NAD^+^ remained stable throughout the incubation period, implying constant availability over the storage period and providing the necessary electron acceptor. Moreover, the reduced lactate and increased pyruvate observed may indicate active oxidation to lactate by the action of LDH-B in the mitochondria or mitochondrial region.

Mitochondrial activity and intracellular Ca^2+^ in live spermatozoa were higher in the G1-10P group. Activation of calcium influx through CatSper channels during capacitation enhances mitochondrial activity (Ferreira et al., 2021), which is essential for sperm hyperactivation and fertilization. Another aspect to underline in our study is the percentage of live spermatozoa with the acrosome reacted. Capacitation, the acrosome reaction, and hyperactivation are processes that involve high amounts of energy and a rewiring of sperm metabolism to meet these requirements (Balbach et al., 2023; Marin-Briggiler et al., 2021; Mohanty et al., 2024; Oppong et al., 2024; Sansegundo et al., 2022; Schmidt et al., 2024; Serafini & O’Flaherty, 2025; Tourmente et al., 2022). Interestingly, we found a higher number of live acrosome-reacted spermatozoa with higher Ca^2+^ in the low glucose extenders, further supporting the hypothesis of an improved metabolic efficiency in this group. High extracellular glucose is a well-known disruptor of Ca^2+^ signaling; high extracellular glucose disrupts calcium signaling in various cell types, contributing to diabetic complications. This occurs through mechanisms like altered calcium influx, release from intracellular stores, impaired removal, and activation of signaling pathways, as detailed in studies exploring the impact on cardiovascular, pancreatic, and other cell functions (Gerbino et al., 2012; Kain et al., 2016; Men et al., 2013; Sun et al., 2019; Tao et al., 2022).

Another important aspect of our study relies on the probable role of aerobic glycolysis and the importance of lactate dehydrogenases in the spermatozoa to sustain sperm functionality (Pena et al., 2024a, 2025). NAD^+^ recycling is likely key for cell survival because many oxidative biochemical pathways produce NADH. Pyruvate supplementation rescues cell metabolism in cells, like spermatozoa in conservation, by restoring NAD^+^/NADH balance (Titov et al., 2016), and the strategy of pyruvate addition to conservation media has been revealed to be successful in numerous reports from different laboratories (Becerro-Rey et al., 2024a, b; Bruemmert et al., 2002; Darr et al., 2016; Gibb et al., 2015; Martin-Cano et al., 2024a); moreover, lactate as the only energy source promotes capacitation in stallion spermatozoa (Hernandez-Aviles et al., 2023; Ramirez-Agamez et al., 2024). A major factor influencing stallion sperm metabolism may relate to the flux under different glucose and pyruvate conditions, which depends on the relative rates of pyruvate reduction to lactate, and lactate oxidation to pyruvate. Stallion spermatozoa have three isoforms of LDH: A, located in the cytoplasm in the acrosomal region, B located in the cytoplasm in the mitochondrial region, and C, located all along the flagella. The existence of a lactate shuttle in the stallion spermatozoa has been recently proposed, based in the intracellular locations of the three isoforms, and the effects of specific inhibitors (Pena et al., 2024a, 2025). Isoforms of LDH are highly compartmentalized in the spermatozoa. LDHA and LDHC expressed in the acrosomal region and tail, respectively, have a higher affinity for pyruvate; these isoenzymes reduce pyruvate to lactate, and the electron donor is NADH, which is oxidized to NAD^+^. NAD^+^ favors glycolysis, supporting glycolytic enzymes in the flagellum and improving sperm velocity. In the mitochondrial region of the cytoplasm, the lactate generated is oxidized to pyruvate by the isoform present, LDHB, with a higher affinity for lactate. This is oxidized to pyruvate, which is imported in the mitochondria and feeds the TCA cycle, providing reducing equivalents and donating electrons to the ETC to generate energy to phosphorylate ADP, producing ATP.

Although our study provides potentially interesting clues, it also has some limitations; the first is the fact that all the extenders contained pyruvate, although overtly distinct concentrations, not providing the highest scenario to disclose pyruvate metabolism on its own. In addition, metabolomics is the trickiest of all the omics, since changes may occur rapidly, and the interpretation of changes in the relative concentration of metabolites has to be interpreted based on their production and consumption (Balbach et al., 2020, 2023; Romarowski et al., 2023). Moreover, real-time metabolic tracing should provide the real scenario of sperm metabolism.

However, despite these limitations, our study suggests that modification of the stallion metabolism through the modification of energy sources in the media may be a strategy to improve sperm conservation and suggests that adaptation of energy sources to the desired outcome i.e. capacitation, is needed. Moreover, research on the specific energetic requirements of specific sperm subpopulations or revealing the causes behind stallion-to-stallion variability may improve current sperm technologies.

## Supplementary Information

Below is the link to the electronic supplementary material.

Supplementary Material 1

Supplementary Material 2

Supplementary Material 3

## Data Availability

The raw data that support the findings of this study are not openly available due to reasons of sensitivity and are available from the corresponding author upon reasonable request.
