# Supplementary material for: Targeted metabolomics reveals the impact of glucose and pyruvate on energy metabolism and storage potential of stallion spermatozoa
Source: Metabolomics. 2026 Mar 28;22(2):45. doi: 10.1007/s11306-025-02393-2 (PMC13032957; doi:10.1007/s11306-025-02393-2)
Supplement: Supplementary file 1 — Supplementary Material 1 [file 11306_2025_2393_MOESM1_ESM.docx]

| **Compound** | **Q1 (m/z)** | **Q3 (m/z)** | **CE (eV)** |
| --- | --- | --- | --- |
| 2-Oxoglutarate | 145 | 145 | 0 |
| Phosphoenolpyruvate | 167 | 79 | 20 |
| 6-Phosphogluconoate | 275 | 97 | 10 |
| 6-Phosphogluconate | 275 | 79 | 40 |
| 6-Phosphogluconolactone | 257 | 257 | 0 |
| Aconitic acid | 173 | 173 | 0 |
| ADP (Adenosine 5'-diphosphate) | 426 | 328 | 16 |
| ADP | 426 | 159 | 28 |
| AMP (Adenosine 5'-monophosphate) | 346 | 97 | 24 |
| AMP | 346 | 79 | 38 |
| ATP (Adenosine 5'-triphosphate) | 506 | 408.1 | 22 |
| ATP | 506 | 159 | 38 |
| Citric acid | 191 | 191 | 0 |
| Citric acid | 191 | 111 | 15 |
| Citric acid | 191 | 87 | 15 |
| D-Fructose 6-phosphate | 259 | 97 | 14 |
| D-Fructose 6-phosphate | 259 | 79 | 48 |
| D-Glucose 6-phosphate | 259 | 97 | 14 |
| D-Glucose 6-phosphate | 259 | 79 | 48 |
| Dihydroxyacetone phosphate | 169 | 169 | 0 |
| Dihydroxyacetone phosphate | 169 | 97 | 10 |
| Dihydroxyacetone phosphate | 169 | 79 | 30 |
| D-Maltose | 341.1 | 179 | 4 |
| D-Maltose | 341.1 | 161 | 5 |
| D-Xylose | 149.05 | 89.02 | 4 |
| D-Xylose | 149.05 | 71.01 | 4 |
| D-Fructose | 179.1 | 89 | 4 |
| D-Fructose | 179.1 | 59.2 | 16 |
| Fructose 1,6-bisphosphate | 339 | 241 | 15 |
| Fructose 1,6-bisphosphate | 339 | 97 | 20 |
| Fumaric acid | 115 | 115 | 0 |
| Glutathione oxidized (GSSG) | 613 | 230 | 40 |
| Glutathione reduced (GSH) | 308 | 179 | 10 |
| Gluconolactone | 177 | 99 | 10 |
| Gluconolactone | 177 | 71 | 16 |
| Glucose/Galactose (isomers) | 179 | 89 | 10 |
| Hydroxyglutaric acid | 147 | 128.9 | 5 |
| Hydroxyglutaric acid | 147 | 85.1 | 15 |
| Inosine 5'-monophosphate (IMP) | 347 | 97 | 22 |
| Inosine 5'-monophosphate (IMP) | 347 | 79 | 44 |
| Itaconic acid | 129 | 129 | 0 |
| Lactate | 89 | 45.3 | 9 |
| Lactate | 89 | 43.3 | 10 |
| Lactose | 341.1 | 179 | 4 |
| Lactose | 341.1 | 161 | 5 |
| L-Arabinose | 149 | 89.1 | 4 |
| L-Arabinose | 149 | 59.2 | 12 |
| L-Aspartic acid | 132 | 88 | 10 |
| L-Aspartic acid | 132 | 71 | 14 |
| L-Glutamic acid | 146 | 128 | 8 |
| L-Glutamic acid | 146 | 102 | 11 |
| L-Glutamine | 145.1 | 127 | 7 |
| L-Glutamine | 145.1 | 109 | 10 |
| L-Malic acid | 133 | 115 | 8 |
| L-Malic acid | 133 | 71.1 | 14 |
| NAD | 662.1 | 539.6 | 20 |
| NAD | 662.1 | 408.1 | 30 |
| NADH | 663.9 | 407.9 | 25 |
| NADP | 742.1 | 619.8 | 15 |
| NADP | 742.1 | 408.1 | 25 |
| NADPH | 744.1 | 663.9 | 25 |
| NADPH | 744.1 | 408.1 | 35 |
| Pyruvate | 87 | 87 | 0 |
