# Supplementary material for: Targeted metabolomics reveals the impact of glucose and pyruvate on energy metabolism and storage potential of stallion spermatozoa
Source: Metabolomics. 2026 Mar 28;22(2):45. doi: 10.1007/s11306-025-02393-2 (PMC13032957; doi:10.1007/s11306-025-02393-2)
Supplement: Supplementary file 3 — Supplementary Material 3 [file 11306_2025_2393_MOESM3_ESM.docx]

Supplemetary figure 1.- .- Glycolysis metabolomics, stallion ejaculates were processed as described in material and methods and stored up to 96 h at r.t. in media with different concentrations of glucose and pyruvate: 1mM glucose one mM pyruvate (1G), 1mM glucose 10 mM pyruvate (1G 10p), 40 mM glucose 1 mM pyruvate (40G), 40 mM glucose 10 mM pyruvate (40G10P), 67mM glucose 1 mM pyruvate (67G) and 67 mM glucose and 10 mM pyruvate (67G10P). Data represent log transformed area under the curve data derived from UHPLC-MS QqQ and are given as means ± SE. Results are derived from 12 replicates, 4 different stallions, 3 replicates each **P*<0.05

Supplementary figure 2.- Pentose phosphate pathway (PPP) metabolomics, stallion ejaculates were processed as described in material and methods and stored up to 96 h at r.t. in media with different concentrations of glucose and pyruvate: 1mM glucose one mM pyruvate (1G), 1mM glucose 10 mM pyruvate (1G 10p), 40 mM glucose 1 mM pyruvate (40G), 40 mM glucose 10 mM pyruvate (40G10P), 67mM glucose 1 mM pyruvate (67G) and 67 mM glucose and 10 mM pyruvate (67G10P). Data represent log-transformed area under the curve data derived from UHPLC-MS QqQ and are given as means ± SE. Results are derived from 12 replicates, 4 different stallions, 3 replicates each.

Supplementary figure 3.- Changes in methylglyoxal and reduced glutathione GSH. stallion ejaculates were processed as described in material and methods and stored up to 96 h at r.t. in media with different concentrations of glucose and pyruvate: 1mM glucose one mM pyruvate (1G), 1mM glucose 10 mM pyruvate (1G 10p), 40 mM glucose 1 mM pyruvate (40G), 40 mM glucose 10 mM pyruvate (40G10P), 67mM glucose 1 mM pyruvate (67G) and 67 mM glucose and 10 mM pyruvate (67G10P). Data represent log-transformed area under the curve data derived from UHPLC-MS QqQ for methylglyoxal, and flow cytometry (relative fluorescence units) for GSH; data are given as means ± SE. Results are derived from 12 replicates, 4 different stallions, and 3 replicates each. * *P*<0.05; ** *P*<0.01
